# Sequential Laser Photoresection and Cryotherapy for Very Late Recurrent Subglottic Atypical Carcinoid: A Case Report

**DOI:** 10.1002/rcr2.70771

**Published:** 2026-09-22

**Authors:** Meridiana Dodaj, Lorenzo Carriera, Pier‐Valerio Mari, Stefano Baglioni, Roberto Lipsi

**Affiliations:** ^1^ Department of Pulmonology and Sub‐Intensive Respiratory Unit Ospedale Santa Maria Della Misericordia Perugia Italy; ^2^ Facoltà di Medicina e Chirurgia Università Cattolica del Sacro Cuore Rome Italy; ^3^ Department of Internal Medicine San Carlo di Nancy Hospital Rome Italy

**Keywords:** atypical carcinoid, cryotherapy, interventional bronchoscopy, laser photoresection, subglottic tumour

## Abstract

Atypical pulmonary carcinoids may recur many years after initial treatment, but very late central‐airway recurrence is uncommon and its optimal endoscopic management is uncertain. We report a 67‐year‐old man with metastatic atypical pulmonary carcinoid who developed recurrent subglottic disease almost 20 years after pulmonary resection. Following mild haemoptysis and inspiratory dyspnoea, bronchoscopy showed progression of a known subglottic lesion causing approximately 80% tracheal obstruction. Laser photoresection achieved rapid debulking, haemostasis and marked airway recanalization. Residual endoluminal disease was treated 5 months later with cryotherapy, resulting in complete endoscopic recanalization. No major complications occurred, symptoms resolved, and follow‐up bronchoscopy at 3 months showed sustained airway patency without significant residual obstruction. This case illustrates the feasibility of a sequential multimodal bronchoscopic approach combining immediate thermal debulking with non‐thermal treatment of residual disease.

## Graphical abstract

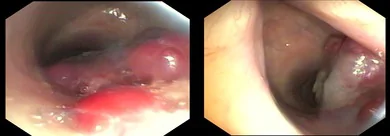

## 1 Introduction

Pulmonary atypical carcinoids are uncommon neuroendocrine neoplasms characterised by an intermediate malignant potential and a greater risk of recurrence and metastatic spread than typical carcinoids. Although their clinical course is often indolent, recurrence may occur many years after initial treatment. Central‐airway involvement of the trachea or subglottic region is particularly uncommon, especially after a prolonged interval from pulmonary resection [1, 2]. Surgical resection remains the preferred treatment for localised or recurrent disease when technically feasible and clinically appropriate. However, interventional bronchoscopy may represent an effective lung‐sparing alternative in selected patients with predominantly endoluminal disease, particularly when surgery is contraindicated because of comorbidities, previous resections, metastatic burden or an unfavourable risk–benefit profile [3, 4, 5]. Among the available modalities, laser photoresection provides immediate tumour debulking, haemostasis and restoration of airway patency, whereas cryotherapy induces delayed cellular necrosis without thermal injury and largely preserves the extracellular matrix and cartilaginous architecture of the airway wall. These complementary properties may support their sequential use. We report the management of a very late recurrent subglottic atypical carcinoid using laser photoresection followed by cryotherapy.

## 2 Case Report

**FIGURE 1:**
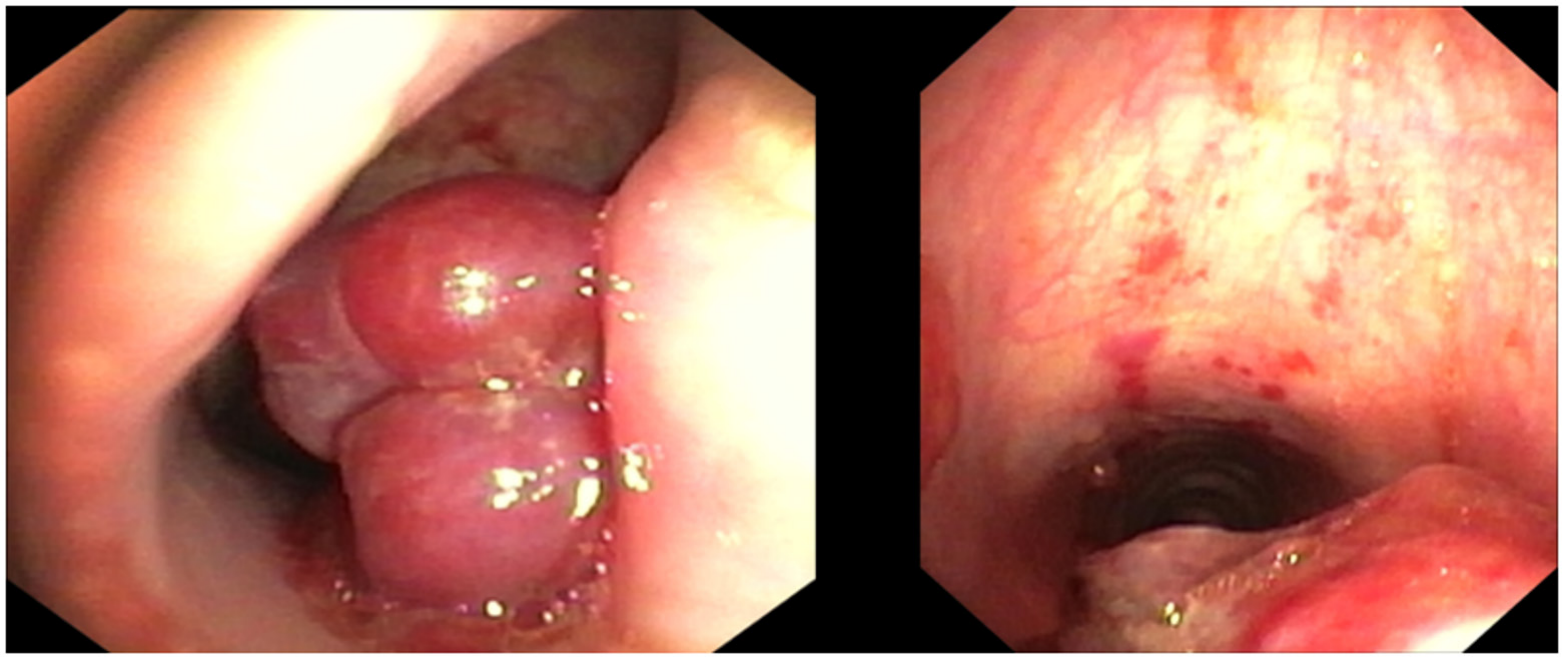
Bronchoscopic appearance and treatment of the subglottic atypical carcinoid in 2021. (A) Vegetating subglottic lesion causing approximately 70% obstruction of the tracheal lumen before laser photoresection. (B) Post‐treatment bronchoscopic appearance showing restoration of airway patency.

**FIGURE 2:**
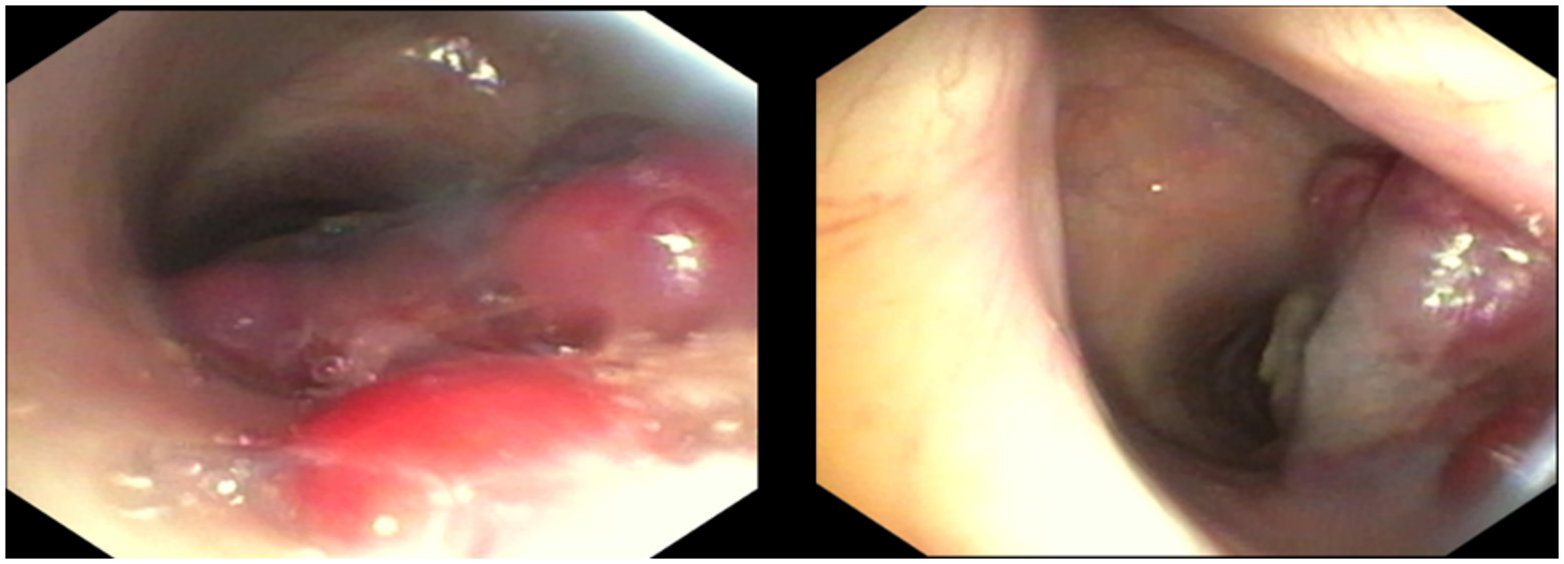
Laser photoresection of recurrent subglottic atypical carcinoid in September 2025. (A) Recurrent endoluminal lesion causing approximately 80% obstruction of the tracheal lumen before treatment. (B) Immediate post‐treatment appearance showing marked airway recanalization with residual endoluminal disease.

**FIGURE 3:**
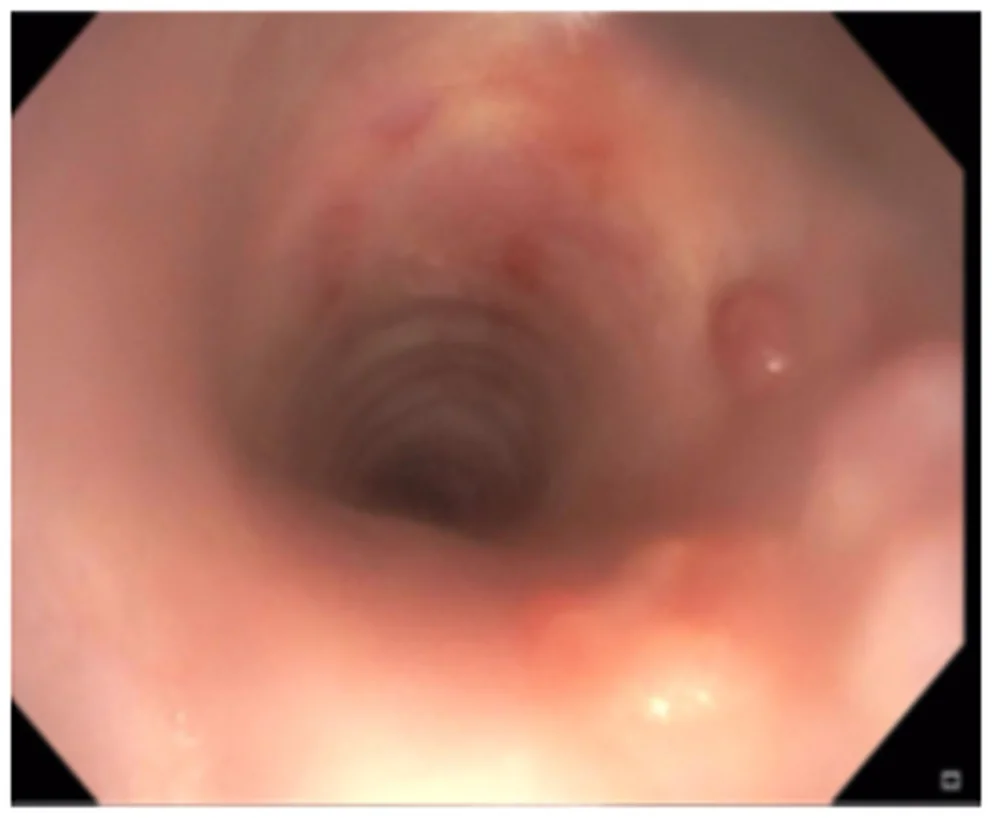
Bronchoscopic follow‐up after cryotherapy. Bronchoscopy performed in May 2026, three months after cryotherapy of the residual subglottic lesion, showed endoscopic recanalization and sustained airway patency without significant residual obstruction. A small mucosal irregularity remained visible at approximately the 4 o'clock position and could not exclude limited persistent superficial disease; no additional biopsy was performed given the already established diagnosis and the successful restoration of airway patency.

A 67‐year‐old former smoker with a 40‐pack‐year smoking history and end‐stage renal disease requiring maintenance haemodialysis presented in August 2025 with intermittent mild haemoptysis and inspiratory dyspnoea. Symptoms developed after the initiation of low‐molecular‐weight heparin for thrombosis following traumatic injury to arteriovenous fistula. Metastatic pulmonary atypical carcinoid with pulmonary and hepatic involvement had been diagnosed in 2006. The patient underwent right middle and lower bilobectomy, followed by long‐term oncological surveillance. Tracheal and hepatic recurrence was documented in 2011. Systemic treatment included octreotide from June 2012 to February 2013, everolimus until 2017, and subsequently lanreotide. Liver‐directed treatments comprised radiofrequency ablation in 2017 and 2020 and transarterial chemoembolisation of a Segment VIII hepatic lesion in 2021. During the same year, flexible bronchoscopy demonstrated a vegetating subglottic lesion causing approximately 70% obstruction of the tracheal lumen. Endoscopic laser photoresection achieved complete relief of the obstruction and restoration of airway patency (Figure 1). The patient subsequently continued clinical, radiological and bronchoscopic surveillance. In March 2025, ^68Ga‐DOTATATE PET/CT demonstrated mild somatostatin receptor‐positive progression of the known subglottic lesion, without evidence of critical airway obstruction. After the onset of haemoptysis and inspiratory dyspnoea, repeat flexible bronchoscopy confirmed progression of the lesion, which caused approximately 80% obstruction of the tracheal lumen. In September 2025, the patient underwent laser photoresection under general anaesthesia via rigid bronchoscopy. The procedure achieved marked airway recanalization, immediate haemostasis and complete resolution of haemoptysis, although residual endoluminal disease remained visible after treatment (Figure 2). In February 2026, the residual lesion was treated with cryotherapy, also performed under general anaesthesia via rigid bronchoscopy. A flexible cryoprobe (2.4 mm in diameter, 900 mm in length; ERBECRYO 2, ERBE, Tübingen, Germany) was introduced through the working channel. Carbon dioxide was used as the cryogen, and the system was operated at Level 2, corresponding to a probe temperature of approximately −41°C to −44°C. Six freeze–thaw cycles were performed, with a freezing time of 15 s per cycle, resulting in complete endoscopic recanalization. No major complications occurred during either procedure, and respiratory symptoms resolved completely. Follow‐up bronchoscopy performed in May 2026 confirmed sustained airway patency without significant residual endoluminal obstruction (Figure 3).

## 3 Discussion

This case highlights the potential for atypical pulmonary carcinoid to recur within the central airways almost two decades after initial pulmonary resection. Although pulmonary carcinoids generally have a more indolent course than other pulmonary neuroendocrine neoplasms, atypical carcinoids retain a clinically meaningful risk of late recurrence and metastatic progression. The present case therefore reinforces the importance of long‐term surveillance in pulmonary carcinoids, particularly in patients with atypical histology and previously recurrent or metastatic disease, consistent with the recently updated ENETS guidance [6]. Interventional bronchoscopy was considered particularly appropriate because the patient had metastatic disease, previous bilobectomy, end‐stage renal disease requiring haemodialysis and a history of multiple systemic and liver‐directed treatments. In this context, further surgery would have entailed substantial risk, whereas a bronchoscopic strategy offered symptom control and preservation of the remaining lung parenchyma. The sequential approach exploited the complementary characteristics of laser photoresection and cryotherapy (Table 1). Laser therapy provides immediate tumour debulking, rapid haemostasis and prompt restoration of airway patency, making it particularly useful for symptomatic, highly vascularised or potentially obstructive lesions. These properties were especially relevant in our patient, who presented with haemoptysis and approximately 80% obstruction of the tracheal lumen. Nevertheless, thermal techniques may be associated with airway‐wall injury, perforation, cicatricial stenosis and airway fire, and complete eradication of a lesion may not always be advisable during a single procedure when preservation of the surrounding structures is a priority. Cryotherapy has a different mechanism of action, producing cellular injury through repeated freezing and thawing. Its effects are generally delayed, but the technique largely preserves the collagen matrix and cartilaginous architecture of the airway wall. It may therefore allow controlled treatment of residual endoluminal disease while avoiding additional thermal exposure. In the present case, laser photoresection first provided rapid recanalization and haemostasis, whereas subsequent cryotherapy targeted the residual lesion and achieved complete endoscopic patency without major procedure‐related morbidity. Evidence supporting multimodal bronchoscopic treatment of recurrent airway carcinoids remains limited to small retrospective series and case reports. Moreover, the relatively short follow‐up after cryotherapy prevents firm conclusions regarding long‐term local control. Nevertheless, this case suggests that sequential laser photoresection and cryotherapy may represent a feasible lung‐sparing strategy in carefully selected patients when immediate airway recanalization and subsequent treatment of residual disease are both required. Longer bronchoscopic and radiological follow‐up will be necessary to assess the durability of the observed response.

**TABLE 1:** Comparison of the principal technical and clinical characteristics of laser photoresection and bronchoscopic cryotherapy in the management of central‐airway lesions.

Characteristic	Nd:YAG laser photoresection	Bronchoscopic cryotherapy
Physical principle	Thermal energy	Extreme cold
Mechanism of action	Tissue vaporisation and coagulation	Freeze‐induced cellular injury and necrosis
Onset of clinical effect	Immediate	Delayed, generally within 48–72 h
Speed of airway recanalization	Very rapid	Moderate unless cryorecanalisation is performed
Haemostatic effect	Immediate and generally effective	Less immediate; may be delayed
Depth of action	Relatively deep and operator dependent	More superficial and controlled
Risk of airway‐wall perforation	Higher because of thermal injury	Lower
Risk of airway fire	Present; requires strict control of FiO2 (generally < 40%)	Absent
Use in highly vascularised lesions	Particularly useful because of its haemostatic effect	Possible, but haemostasis is less immediate
Principal indications	Urgent debulking, haemoptysis, and central‐airway obstruction	Residual or non‐urgent endoluminal disease, friable lesions, granulomas and selected airway stenoses
Effect on airway tissues	Produces thermal injury	Largely preserves the collagen matrix and cartilaginous architecture
Need for rigid bronchoscopy	Frequently required or preferred	Not always required
Principal complications	Bleeding, airway perforation, thermal injury and airway fire	Bleeding, oedema and obstruction from retained necrotic material
Procedural complexity	Generally greater	Generally lower, depending on the technique used
Combination with other techniques	Mechanical debulking, argon plasma coagulation and airway stenting	Cryorecanalisation, argon plasma coagulation and airway stenting
Principal advantage	Immediate restoration of airway patency and haemostasis	Absence of thermal injury and preservation of airway‐wall architecture
Principal limitation	Risk of thermal injury	Delayed treatment effect

Abbreviations: APC, argon plasma coagulation; FiO_2_, fraction of inspired oxygen; Nd:YAG, neodymium‐doped yttrium aluminium garnet.

## Author Contributions

Conceptualization, R.L. and M.D.; Data collection, R.L.; writing – original draft preparation, M.D., L.C., P.‐V.M, S.B., R.L.; writing – review and editing, M.D., L.C., P.‐V.M, S.B., R.L.; supervision, S.B. All authors have read and agreed to the published version of the manuscript.

## Funding

The authors have nothing to report.

## Consent

The authors declare that written informed consent was obtained for the publication of this manuscript and accompanying images using the consent form provided by the Journal.

## Conflicts of Interest

The authors declare no conflicts of interest.

## Data Availability

The data that support the findings of this study are available from the corresponding author upon reasonable request.
